# A TOM1 variant impairs interaction with TOLLIP, autophagosome-lysosome fusion and regulation of innate immunity

**DOI:** 10.1242/dmm.052140

**Published:** 2025-09-30

**Authors:** Heljä K. M. Lång, Tiffany G. Roach, Maarit Hölttä, Salla Keskitalo, Markku Varjosalo, Kaarina Heiskanen, Megan V. Collins, Mikko R. J. Seppänen, Daniel G. S. Capelluto, Elina Ikonen, Samppa J. Ryhänen

**Affiliations:** ^1^ Children's Hospital, University of Helsinki and Helsinki University Hospital, Pediatric Research Center, FI-00290 Helsinki, Finland; ^2^ Department of Anatomy and Stem Cells and Metabolism Research Program, Faculty of Medicine, University of Helsinki, FI-00014 Helsinki, Finland; ^3^ Protein Signaling Domains Laboratory, Department of Biological Sciences, Fralin Life Sciences Institute and Center for Soft Matter and Biological Physics, Virginia Tech, Blacksburg, VA 24061-0477, USA; ^4^ Minerva Foundation Institute for Medical Research, FI-00290 Helsinki, Finland; ^5^ The Institute of Biotechnology, Helsinki Institute of Life Science HiLIFE, University of Helsinki, FI-00014 Helsinki, Finland; ^6^ The Department of Biochemistry and Developmental Biology and Translational Cancer Medicine Program, Faculty of Medicine, University of Helsinki, FIN-00014 Helsinki, Finland

**Keywords:** Autophagy, Inborn errors of immunity, TOM1, TOLLIP

## Abstract

A recently described G307D variant of the endosomal adaptor protein TOM1 causes severe early-onset multiorgan autoimmunity and combined immunodeficiency. By combining biophysical, biochemical and cell culture experiments, we show that the variant causes a defect in the interaction between TOM1 and TOLLIP, another adaptor protein involved in cargo trafficking and regulation of innate immunity. The G307D variant impairs the ability of TOM1 to reduce TOLLIP phosphatidylinositol 3-phosphate binding, an important regulatory mechanism for cargo trafficking commitment for both proteins. Our experiments using *TOM1* G307D patient cells suggested that the variant affects autophagy, seen as an aggravated response to amino acid starvation and accumulation of autophagosomes due to autophagosome-lysosome fusion defect. In addition, inflammatory pathways showed excessive activation in *TOM1* G307D patient cells. Our data suggest that the interaction between TOM1 and TOLLIP has a role in the regulation of the human immune system and highlight the importance of fundamental cellular functions, such as cargo trafficking, in controlling immune responses. Our study also provides insights into the caveats of immunomodulatory and stem cell therapies in patients with TOM1 pathogenic variants.

## Teaser

**Summary:** A TOM1 variant impairs the ability of TOM1 to release TOLLIP from endosomal membranes, leading to reduced clearance of autophagosomes and negative control over inflammatory pathways, and manifesting as a severe phenotype of autoimmunity and immunodeficiency.

## INTRODUCTION

Inborn errors of immunity (IEIs) are conditions caused by monogenic germline variants manifesting as increased susceptibility to infections, autoimmunity, autoinflammation, severe allergy and malignancy. Identification of novel gene defects has broadened our understanding of the mechanisms underlying IEIs (Tangye et al., 2022; Notarangelo et al., 2020). These also include defects in genes functioning outside the classical immunological pathways and affecting, for instance, vesicular trafficking, autophagy and endocytosis (Hait et al., 2020; Watkin et al., 2015; Lopez-Herrera et al., 2012). Treating these patients is particularly challenging as the disease mechanisms are complex and often poorly characterized.

We set out to characterize the mechanism of immune dysregulation found in two related patients harboring a germline heterozygous missense variant (p.Gly307Asp or G307D) in target of Myb1 membrane trafficking protein (*TOM1*) (Keskitalo et al., 2019). The variant is in the GAT domain of *TOM1*, in a locus highly conserved among species, and absent from databases including gnomAD. These patients are the first reported individuals carrying a variant in *TOM1* and presenting with immune dysregulation. The clinical phenotype of the affected mother and her son included childhood-onset multi-organ autoimmunity affecting skin, bowel, lungs and joints, as well as susceptibility to respiratory tract infections. Organ-specific symptoms were accompanied by hypogammaglobulinemia and low counts of dendritic, natural killer and switched memory B cells. In addition, impaired T-cell maturation and regulatory T-cell function were reported in these patients (Keskitalo et al., 2019). Attempts to alleviate the severe autoimmunity with several immunomodulating drugs failed, and the younger patient, with a more severe phenotype, underwent hematopoietic stem cell transplantation (HSCT) but died 6 months post-HSCT owing to lung fibrosis.

TOM1 is a multimodular adaptor protein, which, through interactions with other proteins, including toll-interacting protein (TOLLIP), clathrin and myosin VI, regulates endosomal trafficking and lysosomal degradation of ubiquitinated proteins as well as autophagy (Roach et al., 2021; Burns et al., 2000; Xiao et al., 2015; Tumbarello et al., 2012; Hu et al., 2019). TOM1 has a central GAT domain, through which it interacts with TOLLIP, allowing the recruitment of TOM1 from its primarily cytosolic localization to endosomal membranes (Xiao et al., 2015; Katoh et al., 2004; Yamakami et al., 2003). Through its GAT domain, TOM1 also interacts with ubiquitin, emphasizing its role in protein sorting and degradation (Akutsu et al., 2005). Like TOM1, TOLLIP is modular, containing several domains including a TOM1-binding domain (TBD) and a CUE domain, which functions as a ubiquitin-binding module (Mitra et al., 2013). Previous experiments in mice and *in vitro* have shown that both TOM1 and its binding partner TOLLIP regulate the interleukin-1 receptor (IL-1R) and nuclear factor kappa B (NF-κB) signaling pathways, key mediators of inflammation and innate immunity, and that ablating TOM1 enhances the overall proinflammatory milieu (Burns et al., 2000; Girirajan et al., 2008; Brissoni et al., 2006; Yamakami and Yokosawa, 2004; Martini et al., 2019).

TOM1 has an important role in autophagy, as knocking out TOM1 reduces autophagosomal delivery of endocytic cargo and blocks autophagosome-lysosome fusion (Tumbarello et al., 2012; Hu et al., 2019). Initial experiments involving *TOM1* G307D implied impaired autophagy and inability of patient cells to produce autophagosomes (Keskitalo et al., 2019). Owing to these experimental results, the younger *TOM1* G307D patient was treated with an mTOR inhibitor, everolimus, which, however, led to the worsening of his clinical condition. This unexpected response motivated us to investigate the pathomechanism of the syndrome caused by the *TOM1* G307D variant in more detail by a combination of biophysical, biochemical and cell culture experiments.

## RESULTS

### G307D variant causes local conformational changes in TOM1 but does not affect protein expression level or subcellular localization

**Fig. 1.**
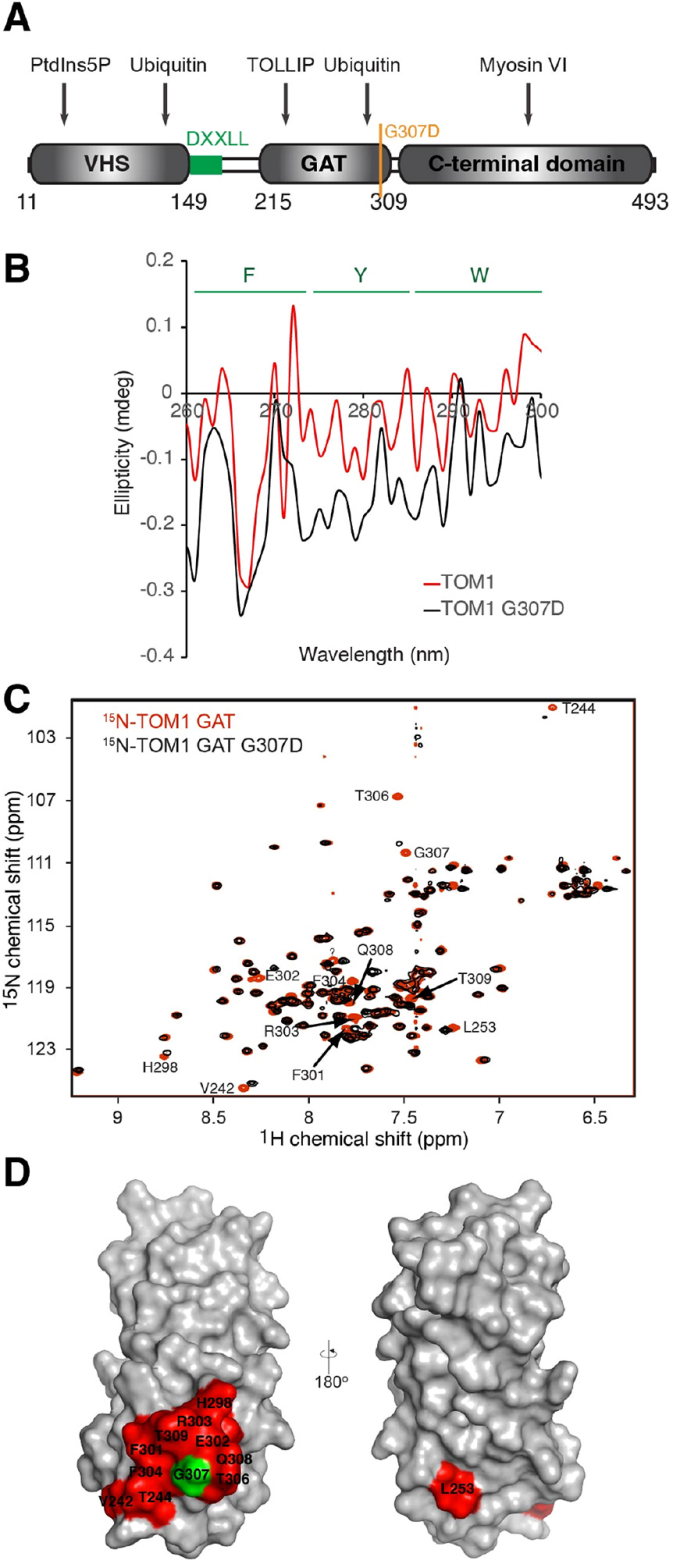
**The G307D variant leads to a local conformational change in TOM1.** (A) Key domains of TOM1 protein, interacting protein regions and position of the G307D residue (yellow). DXXLL (green) is an acidic di-leucine motif found in VHS domain-containing cargo transporters and cargo proteins. (B) Near-UV circular dichroism (CD) spectra of TOM1 (red) and TOM1 G307D (black). Estimated wavelength ranges associated with ellipticity signals from the F, Y and W residues are indicated at the top of the spectra. (C) Overlay of ^1^H, ^15^N heteronuclear single quantum coherence (HSQC) spectra of TOM1 GAT (red) and TOM1 GAT G307D (black). Resonances of the residues displaying major perturbations are labeled. (D) Two surface representations of the TOM1 GAT domain, in which residues exhibiting chemical shift changes in HSQC spectra are colored in red. G307 is colored in green.

The key domains of TOM1 protein, interacting protein regions and position of the G307D residue are depicted in Fig. 1A. To assess the overall impact of G307D on the tertiary structure of TOM1, we collected near-UV circular dichroism (CD) spectra of TOM1 wild-type (WT) and TOM1 G307D. In both cases, negative overlapping absorption bands were observed in regions corresponding to tyrosine and tryptophan residues (Fig. 1B). However, nonoverlapping bands were visualized for the phenylalanine region, suggesting that local conformational changes in TOM1 G307D involve rearrangement in the orientation of phenylalanine residues. The G307D variant is located on the third C-terminal α-helix of the TOM1 GAT domain. Consequently, we collected and overlapped the heteronuclear single quantum coherence (HSQC) spectrum of ^15^N TOM1 GAT with that corresponding to ^15^N TOM1 GAT G307D (Fig. 1C). Chemical shift perturbations only occurred in TOM1 G307D residues around D307, including F301 and F304 (Fig. 1D), consistent with the CD results. These data suggest that the G307D variant leads to local conformational changes in the third α-helix of the GAT domain of TOM1.

Protein expression levels of endogenous TOM1 did not differ between G307D variant patient fibroblasts and control fibroblasts (Fig. S1A,B). TOM1 protein is known to be mainly cytosolic when expressed alone but is recruited to endosomes when co-expressed with TOLLIP (Katoh et al., 2004). Immunofluorescence staining for endogenous TOM1 in patient fibroblasts and control fibroblasts showed similar, cytosolic staining patterns. Also, transient overexpression of TOM1 WT or G307D variant in U2OS cells showed no difference in the subcellular localization of the proteins (Fig. S1C,D).

We treated the proteins with the homobifunctional crosslinkers BS^3^ and glutaraldehyde and found that the G307D variant did not cause oligomerization of TOM1 or changes in heterodimerization of the TOM1-TOLLIP complex (Figs S2 and S3). Unlike TOM1, TOLLIP forms dimers (Figs S2 and S3), in agreement with previous observations, which demonstrated that its CUE domain mediates this process (Mitra et al., 2013).

### G307D variant reduces the inhibitory effects of TOM1 on TOLLIP phosphatidylinositol 3-phosphate binding

Binding of TOM1 to TOLLIP requires the GAT domain (Katoh et al., 2004), and mass spectrometry of overexpressed proteins previously suggested that the G307D variant reduces TOM1 binding to both TOLLIP and ubiquitin (Keskitalo et al., 2019). To investigate whether binding to TOLLIP is disrupted owing to TOM1 G307D variant, we evaluated direct protein binding using surface plasmon resonance with immobilized TOM1 and increasing soluble TOLLIP concentrations. We found no significant differences in binding affinities (Fig. S4A,B and Table S1). TOM1 associates with the TOLLIP TBD and C2 domains via its central GAT domain (Xiao et al., 2015). The G307D variant in TOM1 alters the conformation around α-helix 3 in the GAT domain, potentially affecting the binding site for the TOLLIP C2 domain without compromising its association with the TBD (Fig. 1C; Fig. S5A,B). Given that the GAT domain binds to TOLLIP TBD with ∼300-fold higher affinity than that for the C2 domain (Xiao et al., 2015), the TOM1 variant is unlikely to markedly impact TOLLIP binding, consistent with our findings. To explore whether the G307D variant disrupts interaction of TOM1 with ubiquitin, we used isothermal titration calorimetry (ITC). Although we consistently observed lower enthalpy in the association of TOM1 G307D with ubiquitin compared with that of TOM1 WT, the dissociation constants for both interactions were similar, indicating that G307D variant does not markedly alter the binding affinities between TOM1 and ubiquitin (Fig. S4C and Table S2). Next, we investigated whether differences could be detected using polyubiquitin chains. To address this, we employed fluorescein-labeled K48-linked and K63-linked di-ubiquitin chains, which are primarily associated with proteasomal and endosomal trafficking pathways, respectively. Unlike K48-linked di-ubiquitin, TOM1 was able to bind K63-linked di-ubiquitin, and the G307D variant did not significantly alter this binding (Fig. S6). These results suggest that the conformation of ubiquitin chains, determined by the type of linkage, plays a critical role in TOM1 recognition. Furthermore, the lack of an effect of the G307D variant is consistent with the absence of overlap between the conformational changes induced in the GAT domain and the ubiquitin-binding site within the domain (Fig. 1C; Fig. S5C) (Xiao et al., 2015). Even if the variant were to slightly affect ubiquitin binding, the TOM1 VHS domain also binds ubiquitin with an affinity comparable to that of the GAT domain (Xiong et al., 2024), making it unlikely that the TOM1-ubiquitin interaction would be substantially compromised.

**Fig. 2.**
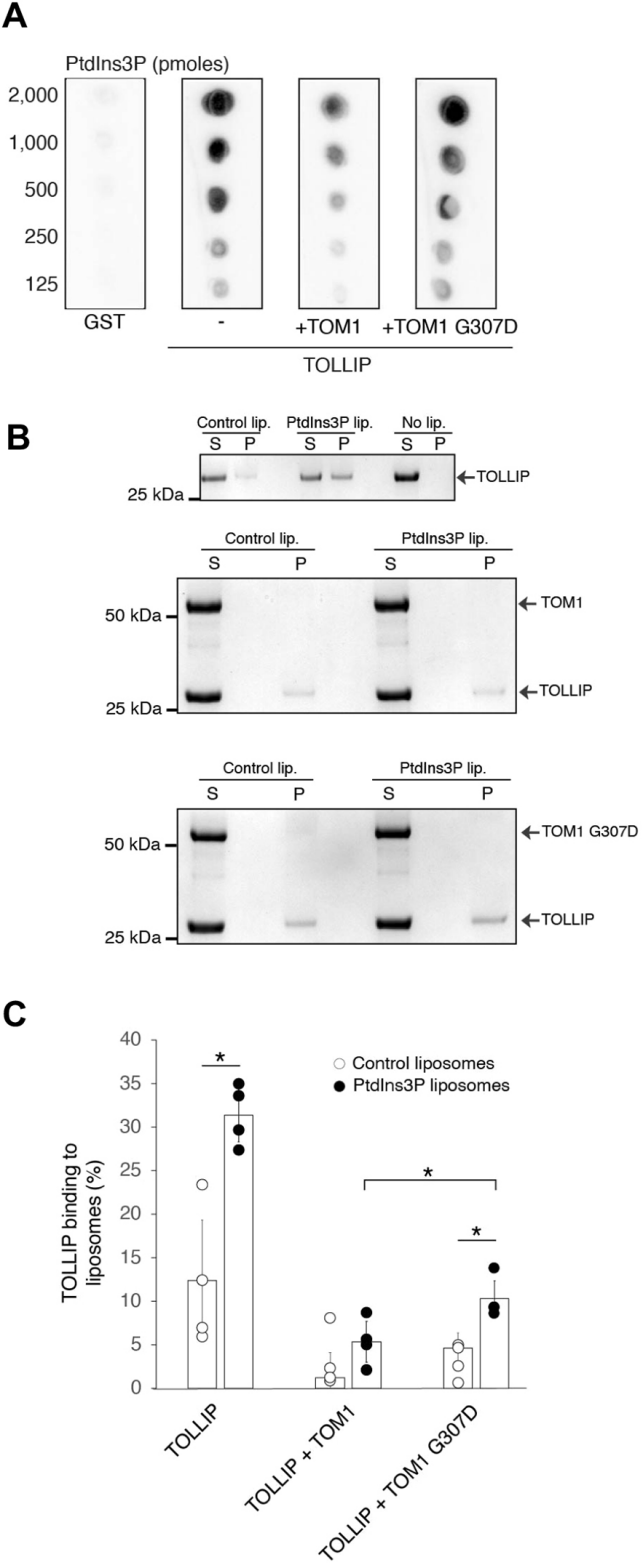
**The G307D mutation in TOM1 reduces the inhibition of TOLLIP binding to phosphatidylinositol 3-phosphate.** (A) Lipid-protein overlay assay showing binding of TOLLIP, in the absence and presence of TOM1 or TOM1 G307D, to phosphatidylinositol 3-phosphate (PtdIns3P). GST was employed as a negative control. (B) Liposome sedimentation assays of TOLLIP, in the absence or presence of TOM1 or TOM1 G307D, with PtdIns3P-free and -containing liposomes. ‘S’ indicates supernatant and ‘P’ indicates pellet. The panel represents the result of at least three independent experiments. (C) Quantifications of the protein band intensities were obtained using ImageJ (RRID:SCR_003070). Data represent the mean±s.d. of four independent experiments. *P=0.034 (unpaired two-tailed Student's *t*-test).

TOM1 binding to TOLLIP decreases the affinity of TOLLIP to phosphatidylinositol 3-phosphate (PtdIns3P), increasing the commitment of both proteins to cargo trafficking (Xiao et al., 2015). Lipid-protein overlay assays showed that the TOM1 G307D variant reduces this inhibitory effect (Fig. 2A). To further evaluate this observation, purified TOM1 was preincubated with TOLLIP and PtdIns3P liposomes. As expected, TOM1 WT significantly reduced TOLLIP binding to PtdIns3P (Fig. 2B). However, TOM1 G307D was significantly less efficient in inhibiting TOLLIP phosphoinositide association (Fig. 2C), suggesting that some TOLLIP remains committed to membrane PtdIns3P. Neither TOM1 nor TOM1 G307 were able to bind PtdIns3P-containing liposomes (Fig. S7). Given the broad preference of TOLLIP for phosphoinositides (Ankem et al., 2011), the variant-induced alteration in the ability of TOM1 to inhibit TOLLIP PtdIns3P binding may extend to other TOLLIP-lipid interactions.

**Fig. 3.**
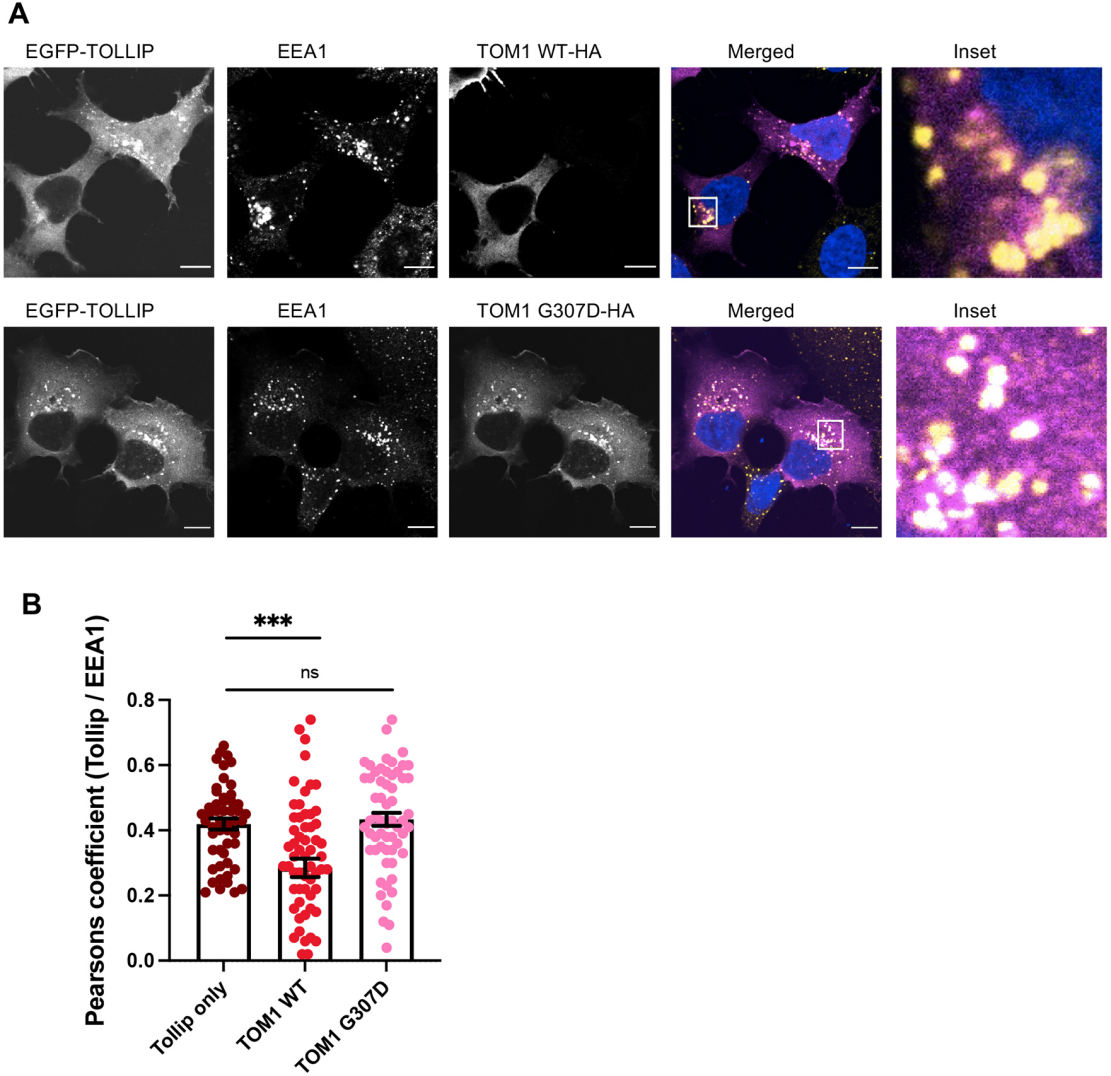
**TOM1 G307D is not able to release TOLLIP from endosomal membranes as efficiently as TOM1 wild type.** (A) Representative images of HEK293A cells stably overexpressing TOLLIP and transiently overexpressing TOM1 wild type (WT) (top row) or G307D variant (bottom row). Additionally, endogenous EEA1 staining is shown. In cells in which TOLLIP and TOM1 WT are co-overexpressed, TOLLIP staining changes from punctate/endosomal to a more cytosolic staining pattern, whereas in cells co-overexpressing TOLLIP and TOM1 G307D, TOLLIP punctate staining pattern prevails. Scale bars: 10 µm. In merged images, EGFP-TOLLIP is shown in magenta and EEA1 in yellow. (B) Quantification of colocalization of TOLLIP and EEA1 in cells overexpressing only TOLLIP, co-overexpressing TOLLIP and TOM1 WT or co-overexpressing TOLLIP and TOM1 G307D. Although overexpression of TOM1 WT reduces TOLLIP colocalization with EEA1 containing early endosomes, overexpression of TOM1 G307D does not. Data represent mean±s.e.m. of pooled data from three independent experiments with 51-62 cells quantified per condition. ns, not significant; ***P=0.0002 (unpaired two-tailed Student's *t*-test).

Next, this observation was assessed in cells. Owing to the lack of a reliable immunofluorescence signal for TOLLIP with the available antibodies, we approached this using overexpression of TOLLIP. In HEK293A cells expressing EGFP-TOLLIP, a punctate staining pattern typical for TOLLIP was observed with confocal microscopy. Consistent with previous reports (Xiao et al., 2015), TOLLIP punctae colocalized with early endosomes marked by the endogenous PtdIns3P binding protein EEA1. In cells co-overexpressing TOM1 WT, the TOLLIP staining pattern became more diffuse and cytosolic, whereas in cells overexpressing TOM1 G307D variant, TOLLIP retained its punctate pattern (Fig. 3A). In cells overexpressing TOM1 WT, colocalization of TOLLIP and EEA1 was significantly reduced, in keeping with previous data showing that TOM1 reduces TOLLIP binding to PtdIns3P. However, in cells overexpressing the G307D variant, TOLLIP and EEA1 colocalization was not reduced (Fig. 3B), in line with the G307D variant impairing the ability of TOM1 to inhibit TOLLIP binding to PtdIns3P (Fig. 2C).

### TOM1 G307D variant affects autophagosome-lysosome fusion in patient fibroblasts

**Fig. 4.**
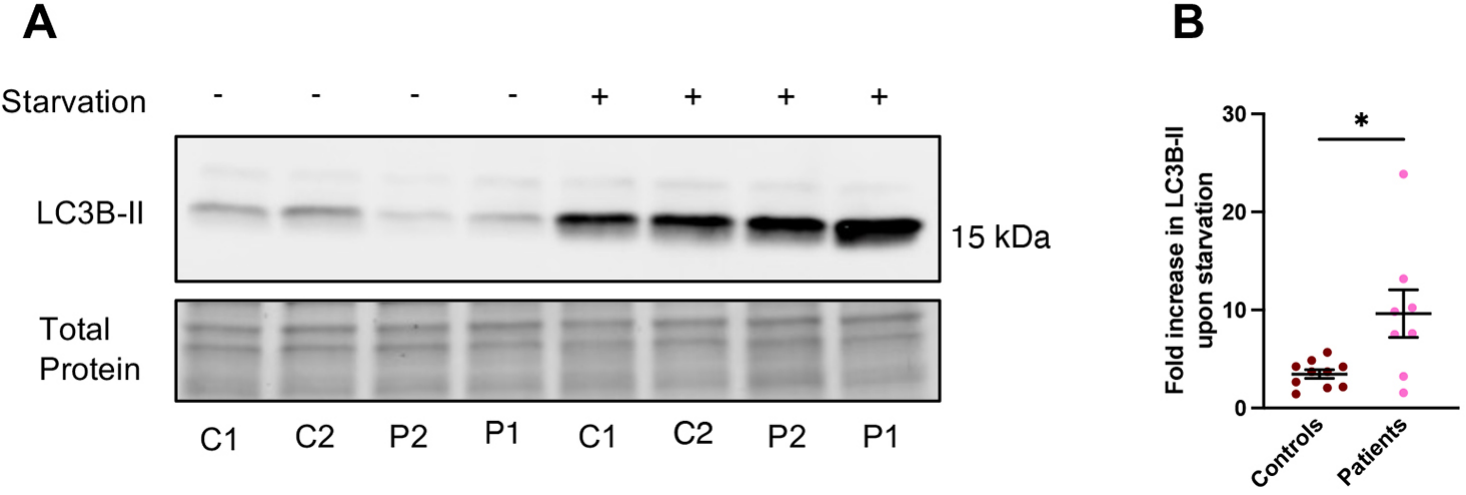
**Patient cells show lower basal autophagy but a more robust response to amino acid deprivation compared to control cells.** Primary skin fibroblasts from two controls (C1, C2) and the two patients (P1, P2) were treated with amino acid starvation [Earle's balanced salt solution (EBSS)] for 2 h to induce autophagy. (A) Representative western blot showing LC3B-II levels before and after EBSS starvation. In cells from both of the patients, LC3B-II levels before EBSS starvation were lower than those in control cells, suggesting lower basal autophagy. (B) Quantification of the western blots revealed that patient cells had a more robust response to EBSS starvation than the control cells, seen as a significantly larger fold change in response to EBSS starvation compared to that of the control cells. Data represent mean±s.e.m. from three independent experiments with duplicate samples in two of the experiments. *P=0.0131 (unpaired two-tailed Student's *t*-test). The quantification of the response to EBSS starvation separately for every cell line is shown in Fig. S8A,B.

Both TOM1 and TOLLIP have critical roles in autophagy (Tumbarello et al., 2012; Gatica et al., 2018). To investigate whether the TOM1 variant affects autophagy, we evaluated the level of autophagy marker protein LC3B (also known as MAP1LC3B)-II in primary skin fibroblasts of two unaffected controls and the *TOM1* G307D patients. Fibroblasts were either kept in complete culture medium or subjected to amino acid starvation, a well-known inducer of autophagy, for 2 h, after which the level of LC3B-II was evaluated using western blotting. The patient cells showed lower LC3B-II levels before Earle's balanced salt solution (EBSS) starvation, suggesting lower basal autophagy compared to that in control cells (Fig. 4A), in line with earlier observations (Keskitalo et al., 2019). However, after 2 h of EBSS starvation, patient cells demonstrated a more robust response to amino acid deprivation than that of control cells, evident as significantly higher fold change in the levels of LC3B-II (Fig. 4B).

**Fig. 5.**
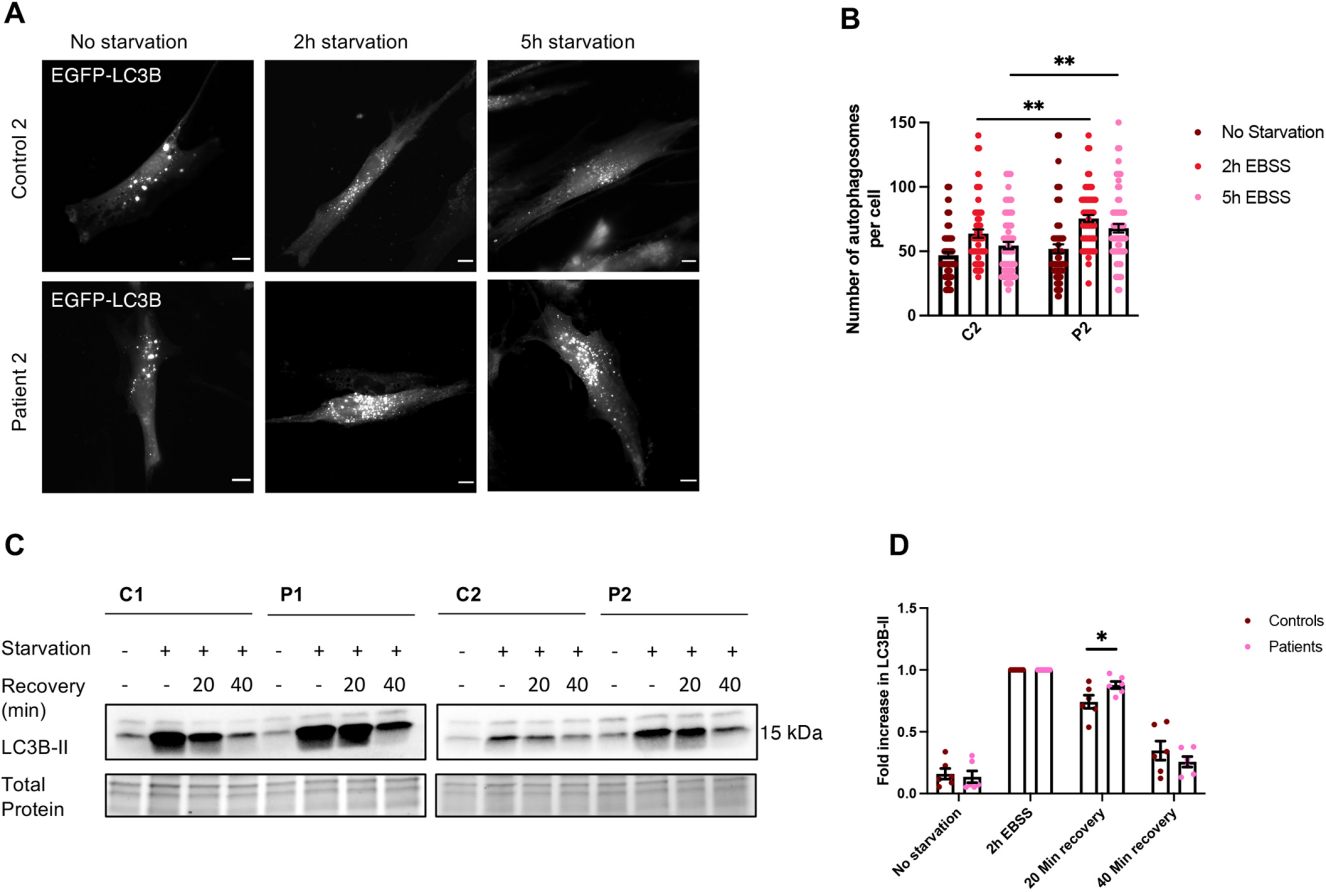
**After amino acid starvation, TOM1 G307D patient fibroblasts show a higher number of autophagosomes and delayed autophagosome clearance compared to control cells.** Skin fibroblasts from patient 2 (P2) and a healthy control (C2) were transiently transfected with an EGFP-LC3B construct to visualize autophagosomes and incubated in complete medium (no starvation) or EBSS for 2 or 5 h (starvation). (A) Representative images of patient and control fibroblasts in which autophagosomes are seen as fluorescent dots. Scale bars: 10 µm. (B) Quantification of the number of autophagosomes. Patient cells showed a larger number of autophagosomes after 2 h of EBSS starvation (**P=0.0067), and, after 5 h of EBSS starvation, the amount of autophagosomes remained significantly higher than that of control cells (**P=0.0027). For each time point, on average 68 cells were imaged and analyzed. Data represent mean±s.e.m. of pooled data from three independent experiments (unpaired two-tailed Student's *t*-test). One datapoint is outside the axis limits and not shown. (C) Skin fibroblasts from both patients (P1 and P2) and two unaffected controls (C1 and C2) were starved (EBSS) for 2 h and then returned to complete medium (recovery) for 20 or 40 min. Representative western blots are shown. (D) Quantification of western blots show significantly higher LC3B-II levels in patient cells compared to those in control cells after a 20 min recovery time. Data represent mean±s.e.m. from three independent experiments with duplicate samples. *P=0.048 (unpaired two-tailed Student's *t*-test).

Next, we investigated autophagy in patient cells utilizing fluorescence microscopy. Patient and healthy control fibroblasts were transiently transfected with an EGFP-LC3B construct (Kabeya et al., 2000), allowing visualization of autophagosomes. After transfection, the cells were either kept in complete culture medium or subjected to EBSS starvation for 2 or 5 h, fixed and imaged. In complete culture medium, the number of autophagosomes was similar in patient and control cells; but, after 2 h of EBSS starvation, the patient cells showed a significantly larger number of autophagosomes than control cells. In patient cells, the amount of autophagosomes remained significantly higher even after 5 h of EBSS starvation, whereas in control cells the number of autophagosomes had returned almost to basal level (Fig. 5A,B). These results suggest that, in contrast to the previously reported reduced ability of peripheral blood mononuclear cells to produce autophagosomes (Keskitalo et al., 2019), *TOM1* G307D fibroblasts are able to produce autophagosomes, and the response to amino acid deprivation was actually more robust than that in control cells.

The accumulation of autophagosomes seen in patient cells could either result from increased autophagosome formation or impaired autophagosome clearance. To evaluate these options, we performed an autophagosome clearance assay as previously described (Tatti et al., 2012). Control and patient primary skin fibroblasts were transferred to EBSS to induce autophagosome formation. After 2 h, the fibroblasts were returned to complete culture medium and allowed to recover for 20 or 40 min. In control cells, restoration of complete culture medium resulted in a decline in LC3B-II levels after a 20 min recovery, as expected (Fig. 5C). In patient cells, however, LC3B-II levels remained significantly higher than those in control cells after a 20 min recovery time (Fig. 5D). This suggests a delayed autophagosome clearance rather than enhanced autophagosome formation in *TOM1* G307D fibroblasts.

**Fig. 6.**
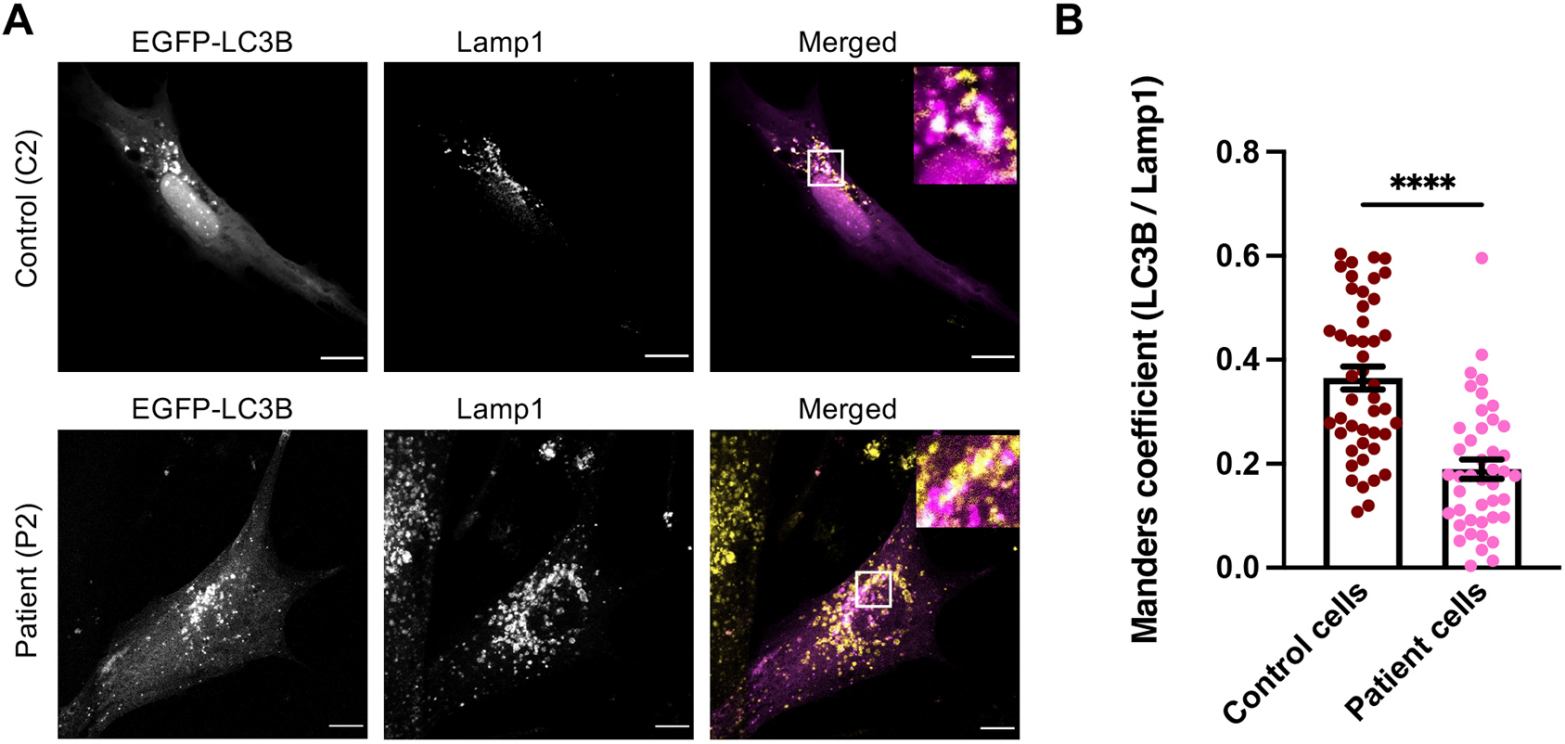
**LC3B and LAMP1 colocalization is reduced in TOM1 G307D patient fibroblasts.** Primary skin fibroblasts from patient 2 (P2) and an unaffected control (C2) were transfected with EGFP-LC3B, starved (EBSS) for 2 h and then stained for lysosomal marker LAMP1 and imaged using confocal microscopy. (A) Representative images of EGFP-LC3B-expressing cells are shown. Scale bars: 10 µm. In merged images, EGFP-LC3B is shown in magenta and LAMP1 in yellow. (B) Quantification of EGFP-LC3B and LAMP1 colocalization. Data represent mean±s.e.m. of pooled data from two independent experiments in which 46 control cells and 43 patient cells were imaged and analyzed. ****P≤0.0001 (unpaired two-tailed Student's *t*-test).

As TOM1 has been shown to be important in the autophagosome-lysosome fusion step (Tumbarello et al., 2012), we hypothesized that the failure of autophagosomes to fuse with lysosomes causes the accumulation of autophagosomes observed in *TOM1* G307D patient cells. To investigate this possibility, we transiently transfected *TOM1* G307D patient fibroblasts and control fibroblasts with the EGFP-LC3B construct and subjected the cells to EBSS starvation for 2 h to induce autophagosome formation. After starvation, cells were fixed and stained for endogenous LAMP1, a lysosomal marker, and imaged using a confocal microscope. In *TOM1* G307D patient fibroblasts, we found that the colocalization of LC3B and LAMP1 was significantly reduced to 54% of the colocalization of LC3B and LAMP1 in healthy control cells (Fig. 6A,B). These data suggest that the G307D variant in TOM1 interferes with autophagosome-lysosome fusion at the late stages of autophagy, providing a plausible explanation for the observed accumulation of autophagosomes and the robust elevation of LC3B-II levels after EBSS starvation (Figs 4 and 5). This hypothesis is supported by proximity labeling mass spectrometry data, which revealed that protein interaction between TOM1 G307D variant and myosin VI was reduced to 69% compared to the interaction between TOM1 WT and myosin VI (Fig. S9).

### TOM1 G307D variant causes dysregulation of inflammatory pathways in patient fibroblasts

**Fig. 7.**
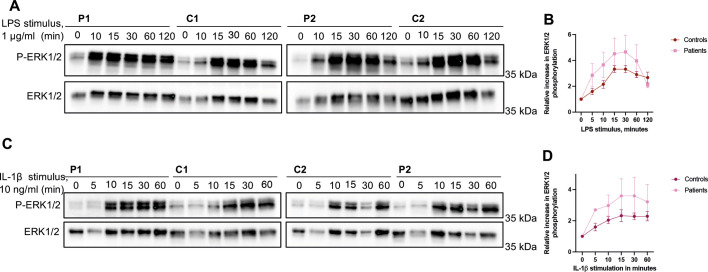
**Patient cells show more robust ERK1/2 phosphorylation after LPS or IL-1β stimulation compared to healthy control cells. (**A) Representative western blots of fibroblasts stimulated with LPS (1 µg/ml) for the indicated times. (B) Quantification of phosphorylated (p)-ERK1/2 ratio to total ERK1/2 after LPS stimulation. Patients and controls are pooled together and normalized to unstimulated control. Data represent results from three independent experiments with duplicate samples. (C) Representative western blots of fibroblasts stimulated with IL-1β (10 ng/ml) for the indicated times. (D) Quantification of p-ERK1/2 ratio to total ERK1/2 after IL-1β stimulation. Patients and controls are pooled together and normalized to unstimulated control. Data represent mean±s.e.m. from three independent experiments with duplicate samples.

Both TOM1 and TOLLIP have been shown to have a suppressive function on Toll-like receptor (TLR), IL-1R and NF-κB signaling pathways (Katoh et al., 2004; Girirajan et al., 2008; Brissoni et al., 2006). Because the *TOM1* G307D patients experienced symptoms of immune dysregulation and severe autoimmunity, we hypothesized that the *TOM1* G307D variant causes impairment of these downregulating functions, leading to uncontrolled immune responses. We therefore investigated how the G307D variant affects some of the key immunological pathways in primary skin fibroblasts. We treated control and patient primary skin fibroblasts with LPS to evaluate the TLR4 signaling pathway or with IL-1β to evaluate IL-1R signaling. We noticed that phosphorylation of both ERK1/2 (also known as MAPK3/1) and AKT appeared more robust in the patient cells than in the controls (Fig. 7; Fig. S10). However, we did not detect differences in the phosphorylation of IKB-α (also known as NFKBIA), a widely used marker of NF-κB activation (Fig. S11). The observed aggravated phosphorylation response suggests a selective impairment in the negative regulation of signaling pathways in *TOM1* G307D cells, contributing to the diverse autoimmune phenomena seen in these patients.

## DISCUSSION

The present study demonstrates that TOM1 G307D disrupts the regulation that WT TOM1 exerts on TOLLIP by inhibiting its binding to PtdIns3P and suggests, for the first time, the pathophysiological relevance of this process in the human immune system. We earlier elucidated the unique regulatory mechanism by which TOM1 controls TOLLIP lipid binding and thereby cargo trafficking commitment (Xiao et al., 2015). Release of PtdIns3P binding allows TOLLIP to recruit ubiquitinated protein aggregates at LC3B-enriched autophagosomes, facilitating the fusion of autophagosomes with lysosomes (Li et al., 2021). Defects in this process are expected to alter autophagy and intracellular signaling, providing a plausible explanation for the severe autoimmunity and immune dysregulation observed in patients carrying the *TOM1* G307D variant.

A previous report (Keskitalo et al., 2019) proposed that the *TOM1* G307D variant causes a defect in the initiation of autophagy and production of autophagosomes. Our data suggest that the defect in autophagy occurs at a later phase of the process when autophagosomes fuse with lysosomes. It has previously been shown that myosin VI and TOM1 function together in the last steps of autophagy, and that the loss of either protein leads to an accumulation of autophagosomes unable to mature to autolysosomes (Tumbarello et al., 2012). Interestingly, we observed that TOM1 G307 interaction with myosin VI was reduced compared to that for TOM1 WT, which likely also contributes to the impairment in autophagy seen in patient cells. When taking into consideration our recent findings, it is reasonable to assume that treatment with an mTOR inhibitor led to the exacerbation of symptoms by further increasing autophagosome formation without alleviating the defect in autophagosome-lysosome fusion and thus aggravating accumulation of autophagosomes.

Autophagy plays important roles in immunity, from removal of intracellular pathogens to control of pro-inflammatory signaling, and to secretory pathway and lymphocyte development (Levine et al., 2011; Wu and Adamopoulos, 2017). Autophagy modifies the development of both innate and adaptive immunity, as well as balances and reshapes immune activation to avoid excessive inflammation (Deretic and Levine, 2018; Cadwell, 2016). Disturbances in the autophagic process, as suggested in the *TOM1* G307D patients, are likely to alter this delicate balance and may cause excess inflammatory signaling. Indeed, there are examples of other IEIs, such as LRBA deficiency and the recently reported GIMAP6 deficiency, for which the disease pathology is linked to impaired autophagic flux (Lopez-Herrera et al., 2012; Yao et al., 2022). Downstream effects of TOM1 G307D on PtdIns3P, ERK1/2 and AKT are probably underlying the hypogammaglobulinemia in our patients, thus mimicking monogenic IEIs associated with Class IA PI3Kinase molecule variants (Thouenon et al., 2021). TOM1 variants have not yet been added to the International Union of Immunological Societies classification; but, taking into consideration the findings made so far, it is most likely that patients with TOM1 variants represent those with defects in intrinsic and innate immunity. Further studies with larger patient cohorts and different TOM1 variants are needed to increase our understanding of this disease.

The present findings emphasize the need to understand the pathomechanisms of IEIs in detail to obtain optimal treatment decisions for patients. Our results not only provide an explanation for why an mTOR inhibitor worsened the patient's symptoms, but also potentially account for the unfortunate outcome after allogenic HSCT of the younger patient: the patient's fibroblasts have severely impaired regulation of innate immunity pathways, and although immune effector cells can be replaced by healthy ones with HSCT, cells in other tissues cannot. For the *TOM1* G307D variant, this implies that fibroblasts and other cells continued aggravated, poorly controlled immune signaling, which further stimulated the replaced immune effector cells to exert an aggravated response. In the setting of HSCT, toxic stimuli caused by chemotherapeutic agents in the conditioning regimen and infections during the neutropenic phase further activated innate immunity pathways. Impaired inhibition of these pathways in *TOM1* G307D patient fibroblasts likely caused a vicious circle, offering a plausible explanation for the severe lung inflammation and fibrosis to which the patient eventually succumbed. With our current understanding of the *TOM1* G307D variant, either IL-1β antagonists or JAK inhibitors could be potential treatment options. Furthermore, our data suggesting a disturbance in autophagosome-lysosome fusion warrants caution in using mTOR inhibitors in the treatment of patients with TOM1 variants.

One of the limitations of the present study is that only two patients were available for analyses and that both patients belong to the same family. However, since the initial report of these patients (Keskitalo et al., 2019), an unrelated child carrying a pathogenic *TOM1* variant has been reported (Baxter et al., 2022), with a clinical phenotype resembling that of the *TOM1* patients discussed here. It is likely that other patients carrying pathogenic variants in *TOM1* will be found, and further functional assays studying these variants will broaden our understanding of the phenotypic heterogeneity and ensuing immune dysregulation in these patients. Another limitation is that, owing to the restricted amounts of primary cell material available, the number of experiments conducted was limited, and hence statistical data should be considered with caution. Additional studies with a larger patient cohort are needed to further increase our understanding of this complex condition. On the other hand, a strength of the study is that most of the cell biological experiments were performed with primary patient cells, providing the most reliable setting to evaluate the effects of the *TOM1* variant G307D.

### Conclusions

To conclude, we propose a novel pathogenic mechanism by which the pathogenic *TOM1* missense variant Chr22: 35728994G >p.Gly307Asp disrupts the TOM1-TOLLIP interaction and causes the inability of TOM1 to efficiently release TOLLIP from endosomal membranes. This, together with reduced interaction with myosin VI, leads to impaired clearance of autophagosomes and reduced negative control over inflammatory pathways, eventually manifesting as a complex and severe phenotype of autoimmunity and immunodeficiency. This model also provides an explanation for our treatment failures with mTOR inhibitors and HSCT in the *TOM1* G307D patient. Our findings suggest caution when considering HSCT as a treatment option for patients with TOM1 variants and possibly also for other inborn errors similarly affecting the regulation of innate immunity.

## MATERIALS AND METHODS

### Cell culture and transfection

Primary fibroblasts were obtained from skin biopsies of the *TOM1* G307D variant patients (P1 and P2) and the unaffected father of P2 (C2). Primary human fibroblasts (Coriell, GM00323, RRID:CVCL_7280) served as the second control line (C1). Fibroblasts were maintained in minimum essential medium (Gibco) supplemented with 15% fetal bovine serum (FBS; Gibco), penicillin/streptomycin (100 U/ml each, Lonza) and 2 mM L-glutamine (Gibco). U2OS and HEK293 cells were cultured in Dulbecco's modified Eagle's medium (Lonza) supplemented with 10% FBS. Cells were cultivated at 37°C in 5% CO_2_ and regularly tested negative for mycoplasma. Fibroblasts were transfected with X-tremeGENE HD transfection reagent (Sigma-Aldrich) and HEK293 cells with Lipofectamine (Invitrogen, Thermo Fisher Scientific) according to the manufacturers’ protocols. To reduce cell-to-cell variation in overexpression experiments, a semi-stable pool overexpressing TOLLIP in HEK293A cells was created, and cells overexpressing EFGP-TOLLIP were selected by using G418 antibiotic.

### Plasmids

The cDNA of human *TOLLIP* was cloned into a pEGFPc1 plasmid. For the biophysical and biochemical experiments, the cDNAs for human *TOM1* and *TOM1* G307D were cloned into both pET28a and pGEX4T1 vectors; the cDNA for human *TOLLIP* was cloned into a pGEX6P1 vector. The cDNA for human ubiquitin, in a pET24d vector, was a gift from Dr Julie Forman-Kay (University of Toronto, Toronto, Canada). The *TOM1* WT and *TOM1* G307D plasmids used in cell biological experiments were kind gifts from Dr Markku Varjosalo (University of Helsinki, Helsinki, Finland).

### Protein overexpression and purification

Constructs were transformed in Rosetta *Escherichia coli* cells, and proteins were overexpressed and purified as previously reported (Xiao et al., 2015).

### ITC

ITC interactions were evaluated using a Microcal PEAQ instrument (Malvern Panalytical) in 20 mM HEPES (pH 7.3) and 150 mM KCl. To monitor binding of TOM1 to TOLLIP, TOM1 concentrations in the syringe ranged from 25 to 100 µM, with TOLLIP concentrations ranging from 5 to 20 µM. Ratios of 10:1 or 10:5 were used to achieve an appropriate binding curve. Interaction between TOM1 and ubiquitin was measured using a syringe concentration of 1.5 or 5 mM (ubiquitin), while the TOM1 cell concentration was kept at 50 µM, for ratios equaling 100:1 or 50:1. All protein concentrations were determined using a Thermo Fisher Scientific NanoDrop One instrument at an optical density of 280 nm using 340 nm reading for a corrected baseline. The ITC instrument was equilibrated at 25°C and set to a deferential power of 10 (µcal/s). Either 12 or 18 injections of 3 µl were used, with an initial injection of 0.4 µl to ensure injection quality. Binding traces were analyzed with a one-site binding model using MicroCal PEAQ analysis software (Malvern Panalytical). All experiments were performed with at least one duplicate. Heats of dilution were accounted for by titrating the more highly concentrated protein into buffer and subtracting the resulting traces when necessary. If no heat of dilution was detected, experimental traces were analyzed with a fitted offset determined by the analysis software.

### Surface plasmon resonance

Surface plasmon resonance experiments were performed on a Biacore X-100 instrument. His-TOM1 or His-TOM1 G307D was immobilized on a NiD200M chip (Xantec), while TOLLIP was used as the analyte at concentrations of 1-16 nM. Sensorgrams were analyzed using BIAevaluation software (RRID:SCR_015936) with the two-state conformational change model.

### Circular dichroism

A Jasco J-815 Spectropolarimeter with a Peltier temperature-controlled cell holder was used to record near-UV CD measurements, spanning from 250 to 350 nm. His-TOM1 or His-TOM1 G307D (100 µM each) were prepared in 5 mM sodium citrate (pH 7.3) and 50 mM potassium fluoride. A quartz cuvette with a 1 mm path-length was used to collect five accumulated spectra. Bandwidth and data pitch were set to 1 nm, scanning speed to 50 nm/min and digital integration time to 0.5 s. All spectra were corrected with buffer background.

### Nuclear magnetic resonance

TOM1 GAT (residues 215-309) and TOM1 GAT G307D were grown in minimal medium with ^15^N-labeled ammonium chloride (Cambridge Isotope Laboratories). ^15^N-labeled proteins were purified as reported (Xiao et al., 2015). A Bruker Avance III 600 MHz with a TCI Prodigy Probe was used to obtain two-dimensional [^15^N,^1^H]-transverse relaxation optimized spectroscopy (TROSY)-HSQC using 100 µM protein. Data were processed using Topspin 3.5 (RRID:SCR_014227) and analyzed using Sparky software (RRID:SCR_014228).

### Pull-down assay

To monitor ubiquitin binding, fluorescein-labeled K48- and K63-linked di-ubiquitin chains (UBPBio) were employed. Ni-NTA agarose beads were washed three times with wash buffer and resuspended to generate a 50% slurry. For each reaction, 8 µl of bead slurry was combined with either 20 µg His-tagged bait protein, 200 ng fluorescently labeled ligand or both, and the volume was adjusted to 200 µl with wash buffer. Samples were incubated at 4°C for 1 h on a rotating platform. After incubation, beads were washed three times with wash buffer, centrifuging at 10,000 ***g*** for 1 min between washes. Following the final wash, supernatants were carefully removed, and pellets were resuspended in loading buffer. Samples were then analyzed by SDS-PAGE followed by fluorescent imaging at 490 nm.

### Lipid-protein overlay assay

Lipid membranes were made with a serial dilution of dipalmitoyl PtdIns3P (Echelon Biosciences) on polyvinylidene fluoride (PVDF) membranes. Membranes were then blocked with 0.5% fatty-acid free bovine serum albumin (BSA) in 20 mM Tris-HCl (pH 8.0), 150 mM NaCl and 0.1% Tween-20 (TBS-T) for 1 h at room temperature. GST-TOLLIP (60 nM), in the absence and presence of 120 nM of His-TOM1 or His-TOM1 G307D, was added to the blocking solution and incubated overnight at 4°C. Membranes were washed with TBS-T, followed by incubation with a primary antibody for 1 h. Antibodies used are listed below. After another wash, membranes were incubated with a secondary antibody for 1 h and rinsed with TBS-T and TBS before analysis using chemiluminescence. Horseradish peroxidase (HRP) activity was monitored using SuperSignal West Pico PLUS chemiluminescent reagent (Thermo Fisher Scientific) following the manufacturer's instructions and a ChemiDoc MP system; images were analyzed using ImageLab 6.1 software (RRID:SCR_014210).

### Liposome co-sedimentation assay

Liposomes were composed of 1,2-dioleoyl-sn-glycero-3-phosphocholine (DOPC) and 1,2-dioleoyl-sn-glycero-3-phosphoethanolamine (DOPE) (Avanti Polar Lipids) (1:1 ratio) with or without 3% dipalmitoyl PtdIns3P (Echelon Biosciences). Lipid mixtures were dried under N_2_, resuspended at 2 mg/ml and hydrated for 1 h at 42°C. Liposomes were subjected to five freeze/thaw cycles then extruded through a mini-extruder (Avanti Polar Lipids) at 60°C using a 200 nm polycarbonate membrane. Proteins were preincubated for 1 h at room temperature before the addition of liposomes. Each protein/liposome mixture consisted of 300 µl, with a final concentration of 2.5 µM TOLLIP with or without 2.5 µM His-TOM1 or His-TOM1 G307D and incubated for 1 h. Suspensions were centrifuged at 90,000 ***g*** for 1 h. Resultant pellets were resuspended in the same volume of buffer. Samples were loaded on an SDS-PAGE and analyzed with ImageJ software (RRID:SCR_003070). Quantitative variables were indicated as means with s.d. and compared using unpaired two-tailed Student's *t*-test assuming unequal variances.

### Immunofluorescence microscopy

Cells were grown on coverslips, fixed with 4% paraformaldehyde for 15 min, permeabilized with 0.1% Triton X-100 for 10 min and blocked with 10% BSA in PBS for 30min at room temperature. Cells were then stained with primary antibodies for 1 h at 37°C followed by secondary antibodies for 1 h at 37°C. For detailed information on antibodies, please see the ‘Antibodies used’ section. Coverslips were rinsed in H_2_O and mounted on slides using Mowiol Dabco. Cells were imaged using a widefield microscope (Zeiss Axio Imager) or a confocal microscope (Leica TCS SP8 CARS) with a 63× oil objective. Images were quantified using ImageJ FIJI (RRID:SCR_002285). Colocalization was measured using Mander's or Pearson's correlation coefficient. The number of autophagosomes was calculated manually.

### Western blotting

Cells were washed with ice-cold PBS and lysed in lysis buffer 1 or 2 (details in the Supplementary Materials and Methods). Equal amounts of protein were loaded onto mini-protein TGX stain-free gels and transferred onto a PVDF membrane. Membranes were blocked with 1% BSA or 5% skim milk in TBS-T for 30 min and subsequently probed with primary antibodies at 4°C overnight. Antibodies are listed below. After washing with TBS-T, membranes were incubated with secondary antibodies at room temperature for 1 h. Membranes were washed, incubated with ECL Clarity (Bio-Rad, 170-5061) and imaged with a ChemiDoc MD Imaging System (Bio-Rad). Band intensities were analyzed with ImageJ software and normalized to total protein content quantified with the Stain-Free technology (Bio-Rad). Quantification of the visualized bands was carried out by ImageJ software (RRID:SCR_003070) and normalized to total protein content quantified with the Stain-Free technology (Bio-Rad) as this is considered to be more accurate than using standard housekeeping proteins (Mortiz, 2017; Gilda and Gomes, 2013).

For phosphorylation assays, cells were grown on six-well plates to 80% confluency and stimulated with IL-β (R&D Systems, 201-LB) 10 ng/ml or LPS (Sigma-Aldrich, L4391) 1 µg/ml, for the indicated times. Before harvesting, the cells were washed with ice-cold PBS and scraped in SDS boiling buffer (details in the Supplementary Materials and Methods). Lysates were boiled at 98°C for 10 min and cleared by centrifugation. Equal amounts of protein were loaded onto Mini-Protein TGX Stain-Free gels and transferred onto a PVDF membrane. Membranes were blocked with 3% BSA in TBS-T for 45 min and probed with primary antibodies at 4°C overnight. After washing with TBS-T, membranes were incubated with secondary antibodies at room temperature for 1 h. Membranes were washed, incubated with ECL Clarity (Bio-Rad, 170-5061) and imaged with a ChemiDoc MD Imaging System. Subsequently the membranes were rinsed in TBS-T for 15 min and then incubated in stripping solution (details in Supplementary Materials and Methods) for 15 min in 50°C. Membranes were washed three times in TBS-T and blocked with 3% BSA before re-probing with new primary antibodies at 4°C overnight and thereafter processed as described above.

### Generation of inducible Flp-In™ T-REx 293 cell lines

The cDNA constructs containing WT and G307D mutant *TOM1* were ordered as synthetic genes and cloned into pTO_MYC_BirA_c (Heikkinen et al., 2017) destination vector. Generation and culture of Flp-In T-REx 293 (Thermo Fisher Scientific) cell lines was performed as previously described (Varjosalo et al., 2013; Kärpänen et al., 2006).

### Proximity labeling mass spectrometry (BioID)

BioID experiments together with mass spectrometry were performed as previously described (Heikkinen et al., 2017). Briefly, ∼5×10⁷ cells (5×15 cm dishes) in three biological replicates were induced with 2 µg/ml doxycycline and 50 µM biotin for 24 h. After induction, cells were washed, harvested and lysed in 3 ml lysis buffer [50 mM HEPES pH 8.0, 5 mM EDTA, 150 mM NaCl, 50 mM NaF, 0.5% NP-40, 1.5 mM Na_3_VO_4_, 1 mM PMSF, 1× protease inhibitor cocktail (Sigma-Aldrich)] containing 0.1% SDS and 80 U/ml Benzonase Nuclease (Santa Cruz Biotechnology) on ice. Proteins were bound to Strep-Tactin sepharose and Bio-Spin chromatography columns (Bio-Rad) and eluted with D-biotin (Thermo Fisher Scientific). After C18 purification, samples were analyzed with an Orbitrap Elite ETD hybrid mass spectrometer as described previously (Heikkinen et al., 2017). Each sample was analyzed in triplicate.

### Antibodies used

#### Lipid protein overlay assay

Primary antibody: rabbit anti-GST antibody (Proteintech, 10000-0-AP, RRID:AB_11042316, 1:6665), Secondary antibody: anti-rabbit HRP-conjugated antibody (Promega, W4011, RRID:AB_430833, 1:2500).

#### Immunofluorescence

Anti-TOM1 rabbit monoclonal antibody (mAb) (Abcam, ab170928, RRID:AB_2687908, 1:100), anti-EEA1 mouse mAb (BD Biosciences, 610457, RRID:AB_397830, 1:50), anti-HA-Tag rabbit mAb (Cell Signaling Technology, 3724, RRID:AB_1549585, 1:1000), anti-Lamp1 mouse mAb (Developmental Studies Hybridoma Bank, H4A3, RRID:AB_2296838, 1:200).

#### Western blotting

Anti-TOM1 rabbit mAb (Abcam, ab170928, RRID:AB_2687908, 1:5000), anti-LC3B rabbit mAb (Cell Signaling Technology, 3868, RRID:AB_2137707, 1:1000), anti-phosphorylated (p)-p44/42 MAPK (ERK1/2) rabbit polyclonal antibody (Cell Signaling Technology, 9101, RRID:AB_331646, 1:1000), anti-p-Akt (Ser473) rabbit mAb (Cell Signaling Technology, 4060, RRID:AB_2315049, 1:2000), anti-p-IκBα (Ser32) rabbit mAb (Cell Signaling Technology, 2859, RRID:AB_561111, 1:1000), anti-p44/42 MAPK (ERK1/2) rabbit mAb (Cell Signaling Technology, 4695, RRID:AB_390779, 1:1000), anti-AKT (pan) rabbit mAb (Cell Signaling Technology, 4691, RRID:AB_915783, 1:2000).

### Statistics

Statistical analysis and plotting of data were performed using GraphPad Prism Version 10 software (RRID:SCR_002798). The number of replicates (*n*) used for calculating statistics is specified in the figure legends. Unpaired two-tailed Student's *t*-test was used to evaluate statistical significance. Differences were considered statistically significant at *P*<0.05. Results are expressed as mean±s.e.m. of the indicated number of observations unless stated otherwise.

### Study approval

The study was conducted according to the Declaration of Helsinki and was approved by the Helsinki University Hospital Ethics Committee. Written informed consent was obtained from the mother and father, as well as their permission for the child.

## Supplementary Material

10.1242/dmm.052140_sup1 Supplementary information
