# Supplementary material for: A TOM1 variant impairs interaction with TOLLIP, autophagosome-lysosome fusion and regulation of innate immunity
Source: Dis Model Mech. 2025 Sep 30;18(9):dmm052140. doi: 10.1242/dmm.052140 (PMC12519566; doi:10.1242/dmm.052140)
Supplement: Supplementary information [file dmm-18-052140-s1.pdf]

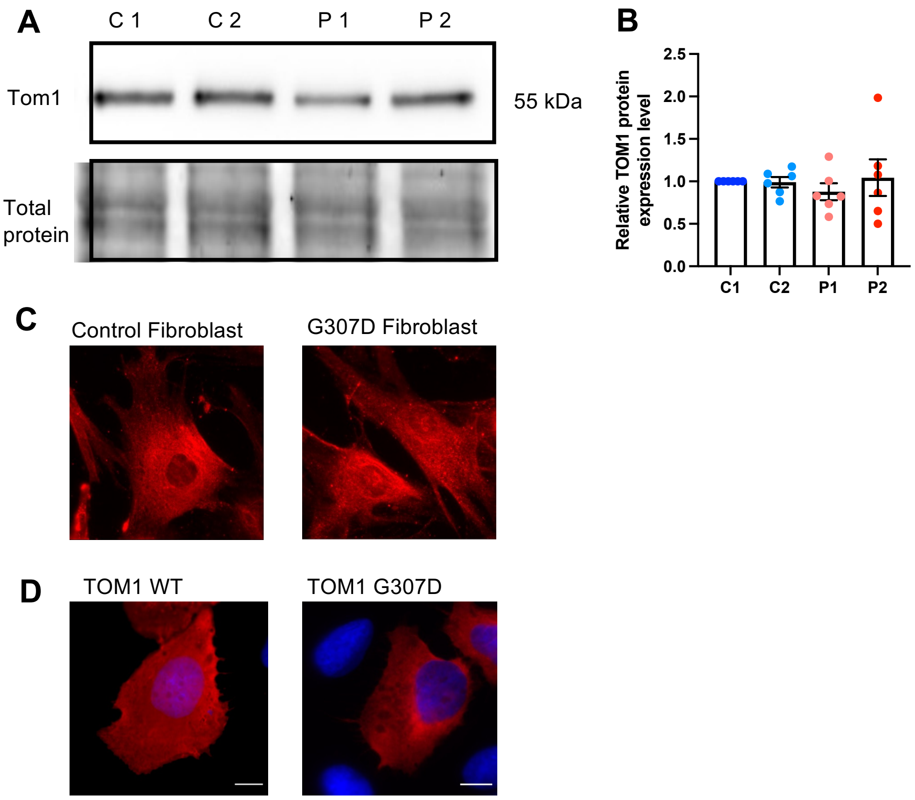

Fig. S1. TOM1 protein levels and subcellular localization do not differ between patient and healthy control cells. (A) TOM1 protein expression levels were evaluated in primary skin fibroblasts of two patients (P1 and P2) and two healthy controls (C1 and C2) using western blot. (B) Quantification of western blot data shows that TOM1 protein expression levels do not differ between patients and healthy controls. Data represent mean ± SEM from three independent experiments with duplicate samples. Band intensities were normalized to C1. (C) Endogenous TOM1 staining in primary skin fibroblasts from a healthy control (C1) and TOM1 G307D patient (P1). (D) Transient overexpression of TOM1 WT or G307D mutant and anti-TOM1 immunostaining in U2OS cells. Scale bar: 10 μm.

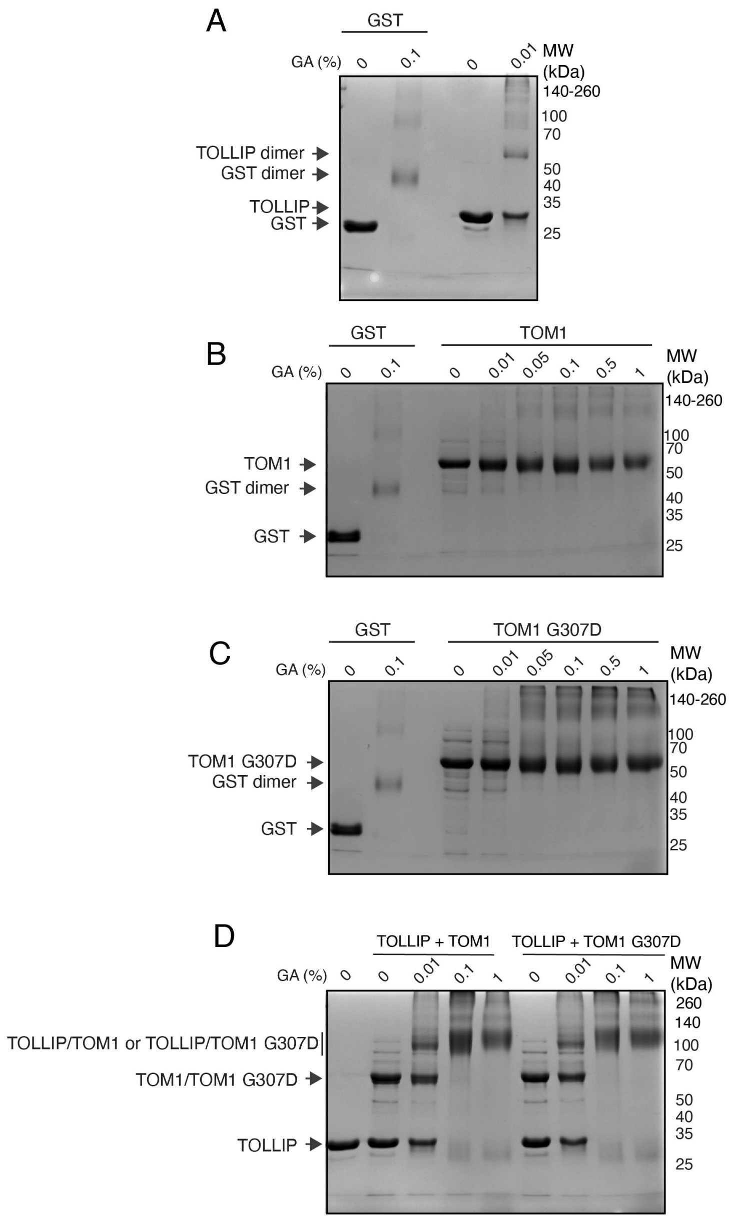

Fig. S2. The G307D mutation in TOM1 does not cause changes in oligomerization. (A) TOLLIP and GST (control) were incubated without and with glutaraldehyde (GA) at the indicated concentrations and oligomer formation was evaluated using SDS-PAGE. (B-C) TOM1 **B**. and TOM1 G307D **C**. were incubated in the absence and presence of the indicated GA concentrations and oligomer formation was tracked using SDS-PAGE. GST was used as a control. (D) TOLLIP was incubated in the absence and presence of either TOM1 or TOM1 G307D at the indicated GA concentrations and the presence of the complexes detected using SDS-PAGE.

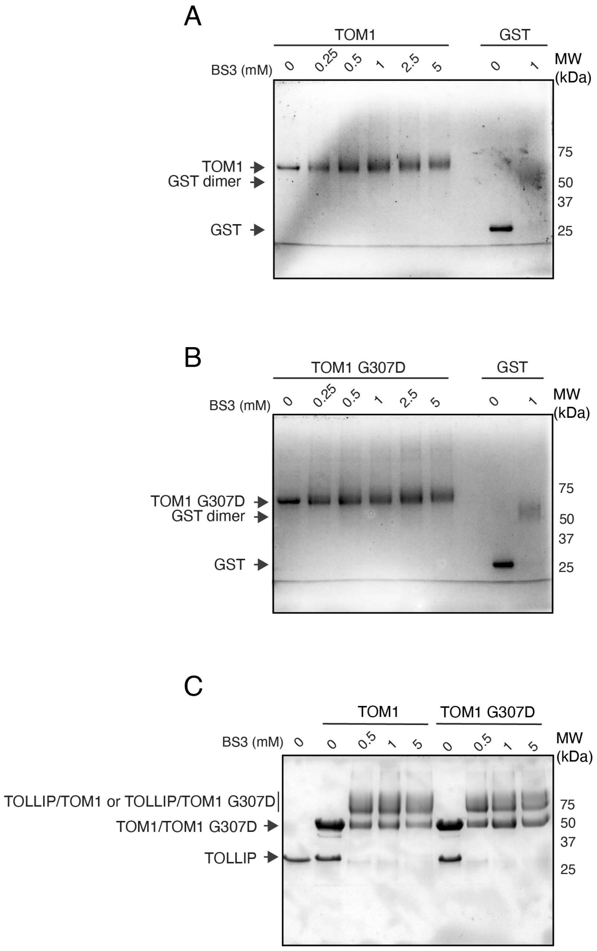

Fig. S3. The G307D mutation in TOM1 does not cause changes in oligomerization. (A-B) TOM1 (A) and TOM1 G307D (**B**) were incubated in the absence and presence of the indicated BS3 concentrations. Oligomer formation was tracked using SDS-PAGE. GST was used as a control. (C**)** TOLLIP was incubated in the absence and presence of either TOM1 or TOM1 G307D at the indicated BS3 concentrations and the presence of the complexes detected using SDS-PAGE.

Disease Models & Mechanisms • Supplementary information

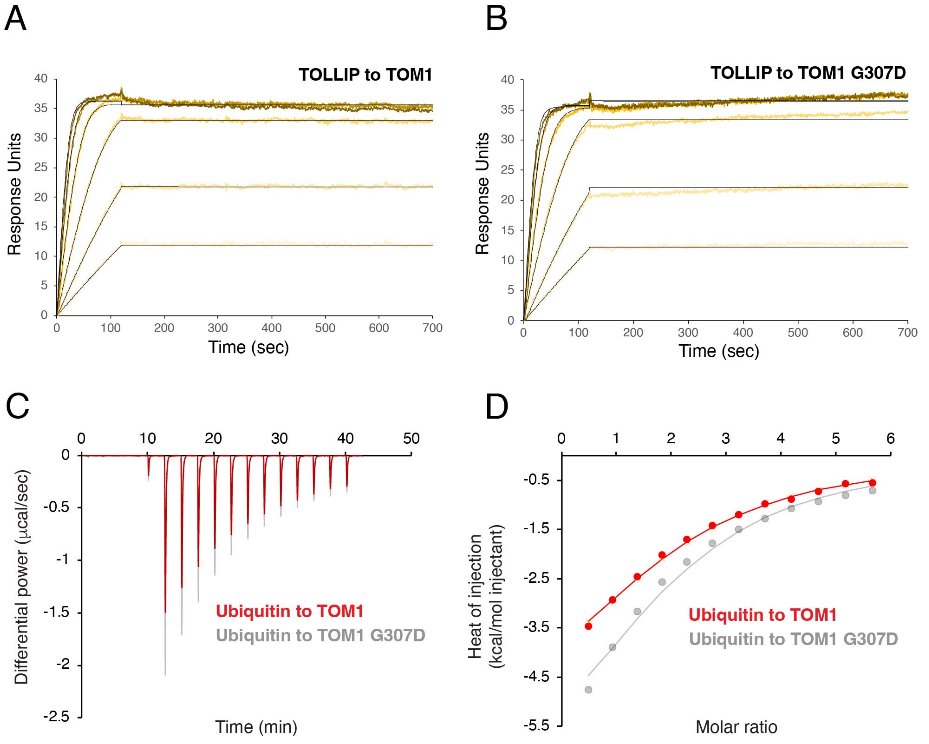

Fig. S4. The G307D mutation in TOM1 does not alter TOLLIP nor ubiquitin binding. (A-B). SPR traces representing the binding of TOM1 (A) and TOM1 G307D (B) to TOLLIP. (C-D) ITC thermograms representing the binding of TOM1 (red) and TOM1 G307D (grey) to ubiquitin. The lines represent the best fit model for 1:1 binding. The data represents 4 independent experiments carried out on TOM1-ubiquitin and TOM1 G307D-ubiquitin interactions.

Disease Models & Mechanisms • Supplementary information

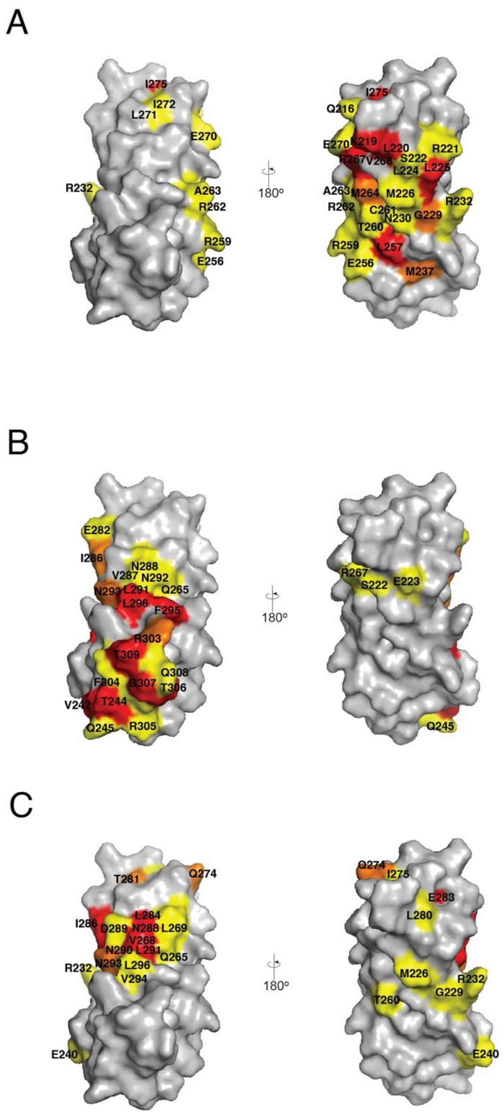

Fig. S5. Protein ligand binding sites in the TOM1 GAT domain. Structural representations of the TOM1 GAT domain highlighting the residues involved in binding with TOLLIP TBD (A), TOLLIP C2 (B), and ubiquitin (C). The relevance of their amino acids is color-coded based on their significance in binding, as determined by NMR experiments. Colors represent the extent of resonance perturbations in the TOM1 GAT domain induced by the ligands: red ($D\delta_{average}$ +1.5 x SD), orange ($D\delta_{average}$ +1 x SD), and yellow ($D\delta_{average}$).

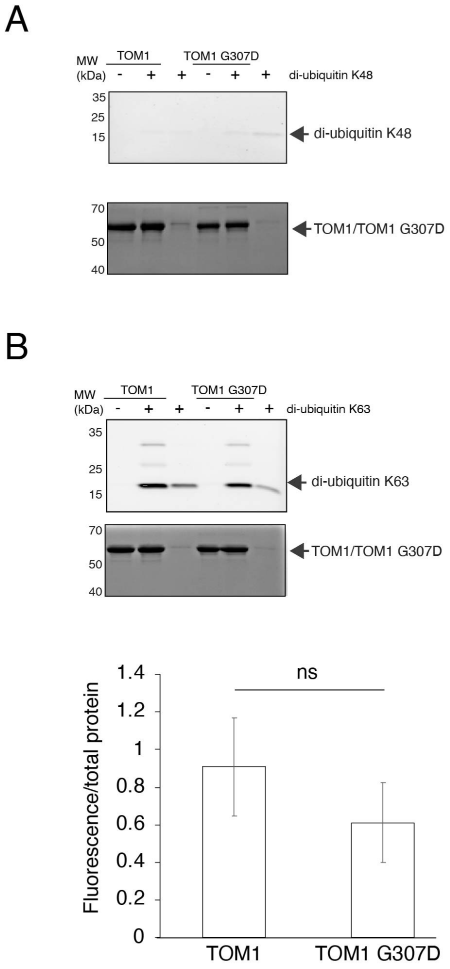

Fig. S6. The G307D mutation in TOM1 does not alter di-ubiquitin-linked interactions. (A) Pull-down assay evaluating the interaction between TOM1 or TOM1 G307D and fluorescein labeled K48-linked di-ubiquitin, excited at 490 nm (top), alongside a Coomassie blue-stained gel showing immobilized His-tagged TOM1 and TOM1 G307D proteins (bottom). (B) Pull-down assay evaluating the interaction between TOM1 or TOM1 G307D and fluorescein labeled K63-linked di-ubiquitin, excited at 490 nm (top), a Coomassie blue-stained gel showing immobilized His-tagged TOM1 and TOM1 G307D proteins (middle), and a histogram quantifying the binding of fluorescently labeled K63-linked di-ubiquitin to TOM1 and TOM1 G307D. Each pull-down assay shown is representative of three independent experiments. p= 0.202; ns, not significant.

Disease Models & Mechanisms • Supplementary information

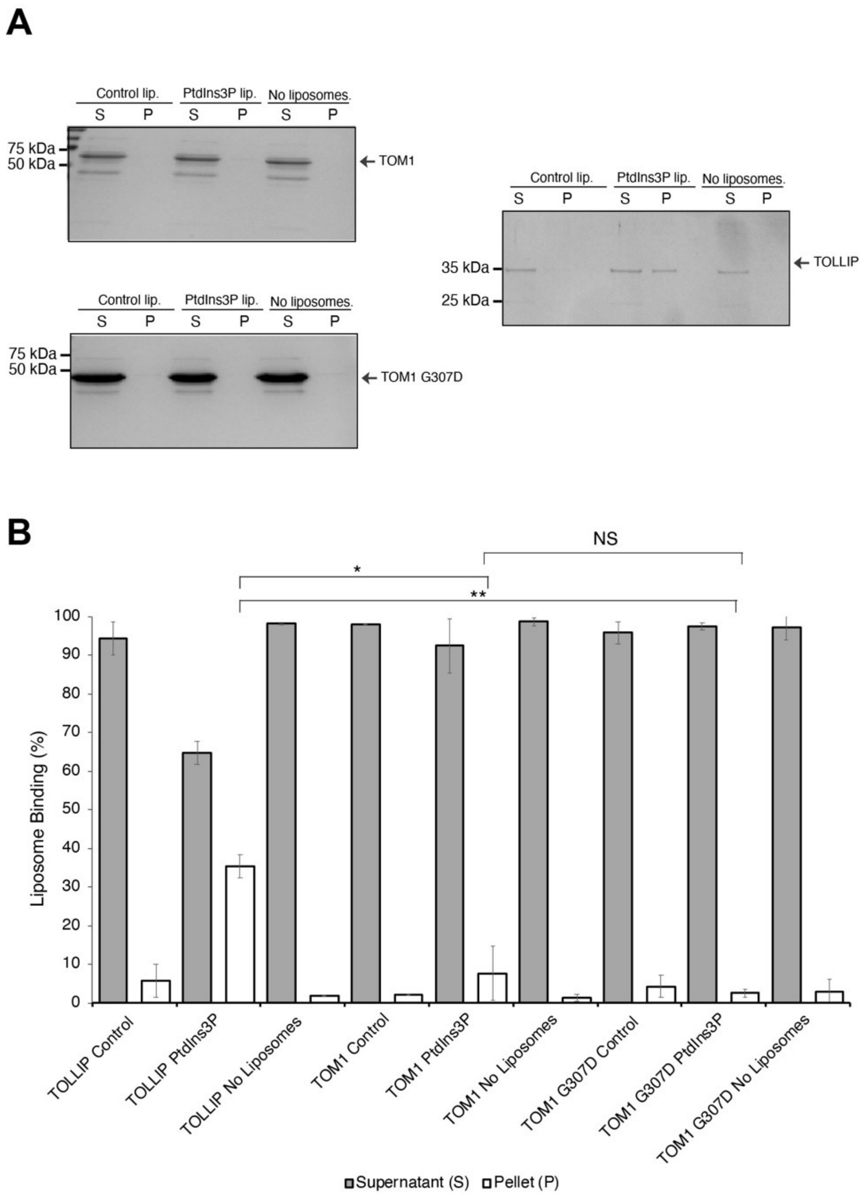

Fig. S7. Nor TOM1 neither TOM1 G307D specifically binds PtdIns3P. (A) Liposome co-sedimentation assay evaluating the assoication of TOM1 (top) and TOM1 G307D (bottom) with PtdIns3P-containing liposomes. TOLLIP was employed as a control. (**B**) Quantification of binding from two independent replicates. * p<0.05; **p<0.005; NS, not significant.

Disease Models & Mechanisms • Supplementary information

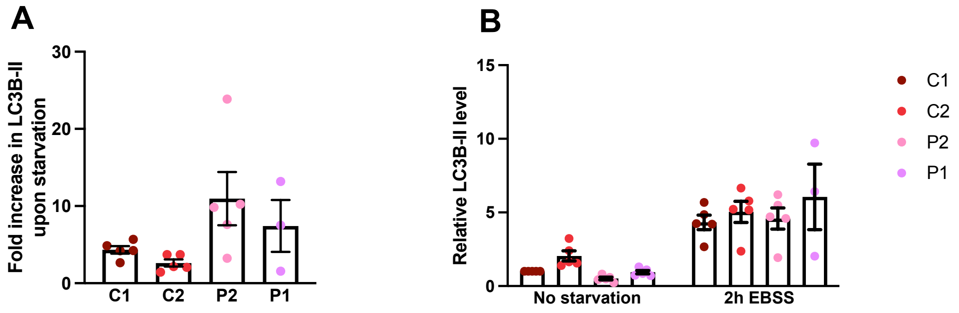

Fig. S8. The respone to EBSS starvation separately for every cell line. Primary skin fibroblasts from two controls (C1, C2) and the two patients (P1, P2) were treated with amino acid starvation (EBSS) for 2 h to induce autophagy. (A) Quantification of western blots shown in figure 4A show that patient cells had a more robust response to EBSS starvation seen as a larger fold increase in LC3B-II levels. (B) Relative levels of LC3B-II before and after EBSS starvation from same data set.

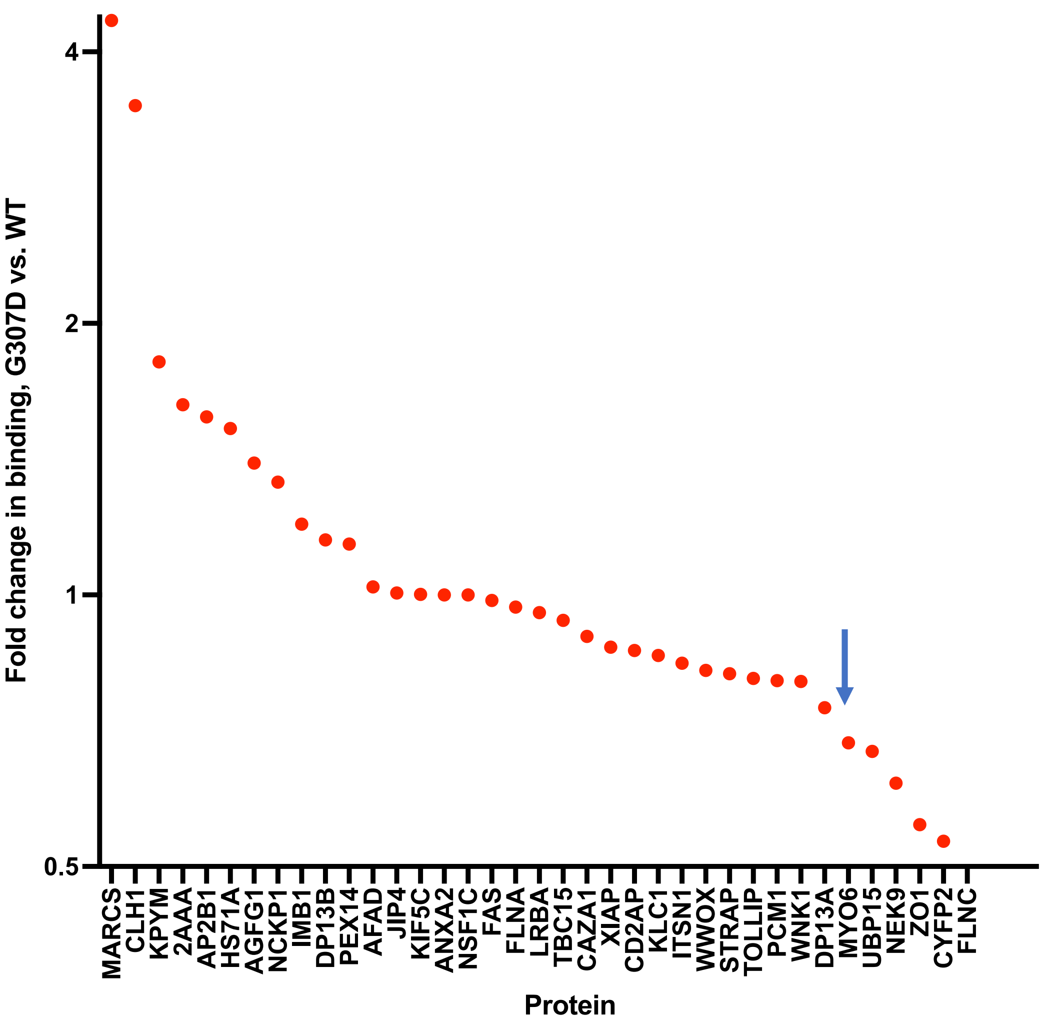

Fig. S9. TOM1 G307D has reduced interaction with several autophagy-linked proteins, including myosin 6 (MYO6) compared to TOM1 WT. Proximity labeling mass spectrometry data with fold-change in protein binding between TOM1 G307D and TOM1 WT shown on the y-axis and different autophagy-related proteins on the x-axis. Interaction between TOM1 G307D variant and myosin VI was reduced to 69% compared to the interaction between TOM1 WT and myosin VI (arrow).

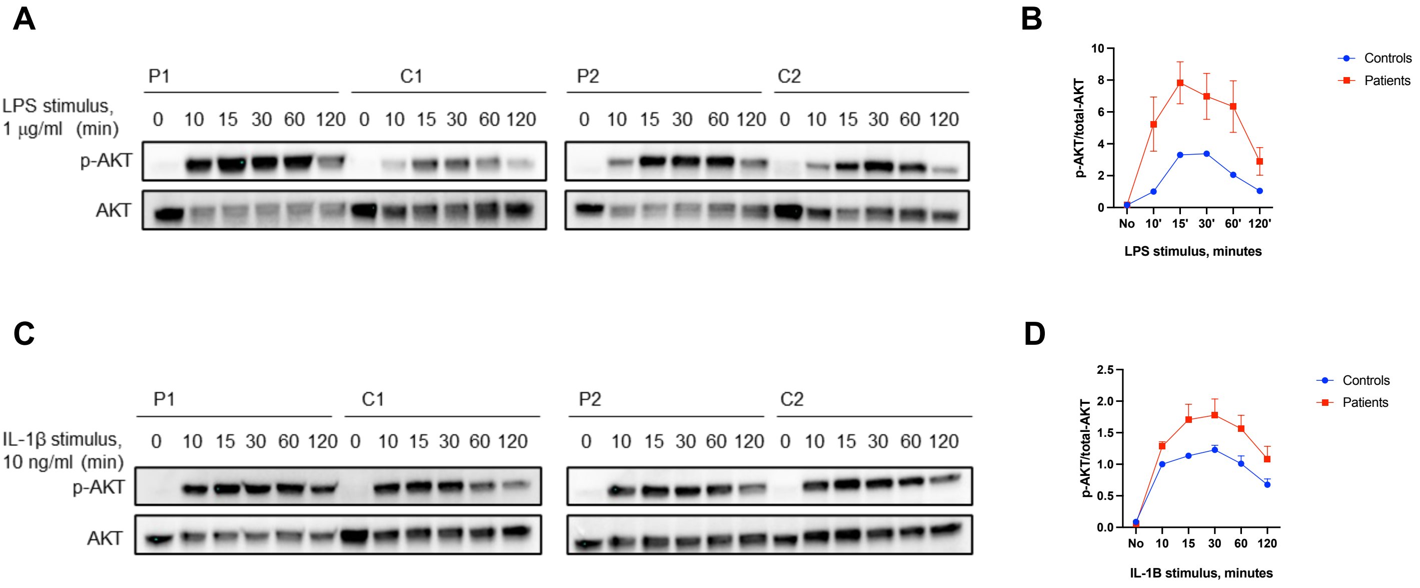

Fig. S10. TOM1 G307D patient cells show a tendency for AKT hyperphosphorylation after LPS or IL-1β stimulation compared to healthy control cells. (A) Representative western blot of fibroblasts after stimulation with LPS (1µg/ml). (B) Quantification of p-AKT ratio to total AKT after LPS stimulation. Patient samples and control samples are pooled together and normalized to control samples 10 min time point. Preliminary data, as data is from one experiment with duplicate samples. (C) Representative western blot of fibroblasts after stimulation with IL-1β (10 ng/ml). (D) Quantification of p-AKT ratio to total AKT after IL-1β stimulation. Patient samples and control samples are pooled together and normalized to control samples 10 min time point. Preliminary data, as data is from one experiment with duplicate samples.

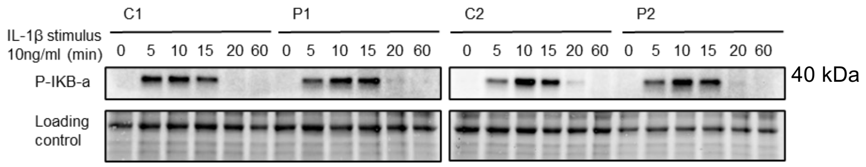

Fig. S11. TOM1 G307D pathogenic variant does not affect IKB-a phosphorylation. Representative western blots of primary skin fibroblasts of patients and controls were stimulated with IL-1β as described previously.

Table S1. Kinetic analysis for the binding of TOM1 and TOM1 G307D to TOLLIP using SPR. Values represent the mean of three independent experiments.

Interaction	K_D (fM)	Chi^2
TOM1-TOLLIP	3.0	0.3
TOM1 G307D-TOLLIP	3.5	0.3

Table S2. Thermodynamic parameters for the binding of TOM1 and TOM1 G307D to ubiquitin using ITC. Values represent the mean of at least three independent experiments. Error margins are displayed as standard deviation values.

Interaction	K_D (µM)	ΔG (kcal. mol^{-1})
TOM1-ubiquitin	77.7±18.8	-5.6±0.1
TOM1 G307D-ubiquitin	104.3±17.0	-5.4±0.1

Disease Models & Mechanisms • Supplementary information

Supplementary Materials and Methods

In-gel crosslinking

Protein oligomerization was evaluated using either bis(sulfosuccinimidyl) suberate (BS^3) (ThermoFisher Scientific) or glutaraldehyde (MP Biomedicals). BS^3 crosslinks ε-amino groups that are 11.4 Å apart, whereas glutaraldehyde covalently associates proteins with a variety of their side chain functional groups, such as amine, phenol, thiol, and imidazole groups [19]. Proteins were incubated with the indicated concentrations of BS^3 for 30 min at room temperature. The same procedure was followed when crosslinking the TOLLIP-TOM1 complexes by preincubating proteins for 30 min before the addition of BS^3. Proteins were also treated with a dilution series of glutaraldehyde and the mixtures incubated for 5 min at 37°C. All samples were run on SDS-PAGE, imaged with a GelDoc Go system (Bio-Rad) and analyzed with ImageLab 6.1 software (RRID:SCR_014210).

Pull-down assay

Fluorescein labeled K48 di-ubiquitin (UBPBio Cat# D1110) and fluorescein labeled K63 di-ubiquitin (UBPBio Cat# 2110)

List of reagents:

Western Blot lysis buffer 1: HEPES (pH 7.4), 1% NP40, 150 mM NaCl, 5 mM EDTA, 25 µg/ml chymostatin, 25 µg/ml leupeptin, 25 µg/ml antipain hydrochloride, 25 µg/ml pepstatin A, 10 mM sodium fluoride, 10 mM orthovanadate, and 1 mM PMSF.

Western Blot lysis buffer 2: RIPA lysis buffer containing 1x TBS, 1% NP-40, 0.1% SDS and supplemented with 10% NaDoc and 1:100 protease inhibitor cocktail (CLAP).

Western Blot lysis buffer 3: SDS boiling buffer containing 2.5% SDS, 250 mM Tris/HCl (pH 6.8), including 50 mM NaF, 10 mM, β-glycerophosphate, 0.5 mM DTT, and 0.5 mM PMSF.

Stripping solution: 10% SDS 20%, 2% 3 M Tris (pH 6.8), and 0.8% 2-mercaptoethanol.

HNN lysis buffer: 50 mM HEPES pH 8.0, 150 mM NaCl, 5 mM EDTA, 0.5% NP-40, 50 mM NaF, 1.5 mM Na3VO4, 1.0 mM PMSF (phenylmethanesulfonylfluoride) and 10 µl/ml protease inhibitor cocktail (Sigma).
